# Physical and mental health conditions account for variability in awareness of age-related changes

**DOI:** 10.3389/fpsyt.2023.1152177

**Published:** 2023-07-19

**Authors:** Serena Sabatini, Shelbie Turner, Helen Brooker, Clive Ballard, Anne Corbett, Adam Hampshire

**Affiliations:** ^1^School of Medicine, Institute of Mental Health, University of Nottingham, Nottingham, United Kingdom; ^2^Division of Geriatrics and Palliative Medicine, Weill Cornell Medical College, New York City, NY, United States; ^3^University of Exeter Medical School, Exeter, United Kingdom; ^4^Ecog Pro Ltd, Bristol, United Kingdom; ^5^Social, Genetic, and Developmental Psychiatry Centre, King’s College London, London, United Kingdom

**Keywords:** views on aging, subjective aging, mental illnesses, diagnosis, chronic health conditions

## Abstract

**Background:**

The concept of Awareness of Age-Related Changes captures people’s perceptions of the positive (AARC-gains) and negative (AARC-losses) age-related changes they experience in several life domains, including their health. We investigated the cross-sectional associations of number and type of physical and mental health conditions with AARC-gains and AARC-losses.

**Methods:**

The sample comprised 3,786 middle-aged and older adults (mean age = 67.04 years; SD = 6.88) participating to the UK PROTECT study. We used hierarchical regression models to analyze whether after having included sociodemographic variables (model 1), number of physical (model 2) and of mental (model 3) health conditions explained a significant additional amount of variance in AARC-gains and AARC-losses, and whether the association between number of conditions and AARC depended on participants’ age. We used multiple regression models to analyze the associations of types of physical and mental health conditions with AARC-gains and AARC-losses.

**Results:**

A higher number of physical health conditions was associated with higher AARC-gains and higher AARC-losses, but the association did not depend on participant age. After controlling for the number of physical health conditions, a higher number of mental health conditions was associated with higher AARC-losses but not with AARC-gains, and the association was stronger among older participants. Small effects were found between greater AARC-gains and current cancer and between greater AARC-losses and diagnoses of mild cognitive impairment, Parkinson’s disease, arthritic condition, cancer in full remission, osteoporosis, depression, anxiety disorders, and personality disorder. The remaining health conditions were either negligibly or non-statistically related to AARC-losses.

**Conclusion:**

Middle-aged and older adults having more physical health conditions and more mental health conditions may be at higher risk of negative views on their own aging. However, specific physical health conditions, such as arthritis, and certain mental health conditions, such as depression, may make adults particularly vulnerable to negative age-related perceptions.

## Introduction

Views on aging (VoA) are an important part of developmental regulation and health behavior, especially in midlife and later life. People with more positive VoA are more likely to implement adaptive strategies in the face of age-related challenges (1). These adaptive strategies often include engaging in health-promoting behaviors such as physical activity (2–7). Because of the strong connections between positive VoA and healthy behavioral regulation, more positive VoA are related to better longer-term physical and mental health (8–10). Conversely, people with more negative VoA are less engaged in health-enhancing and adaptive behaviors and are at greater risk of poorer current and future health, including increased risk of mortality (5, 11–14). The promotion of positive VoA, and the decrease of negative VoA, are therefore increasingly recognized as a useful strategy for physical and mental health promotion (15).

Theoretical models of VoA, including the theoretical model of awareness of age-related change (16, 17), posit that although people’s VoA may have an effect on their future physical and mental health, people’s health history and health status can also act as distal antecedents that shape their VoA. People with existing health conditions may have worse VoA; these more negative VoA, in turn, may threaten their positive health behavior regulation. Given the high prevalence of chronic conditions, it is necessary to consider whether people’s VoA are connected to their experiences of specific chronic conditions and multimorbidities. Recent evidence suggests that in England, about 44, 64, and 90% of people aged 40, 60, and 80 years, respectively, have one or more long-term conditions (18, 19).

### Views on aging and health conditions

Much of the existing literature focuses on how VoA are associated with health behavior, such as physical activity, and longer-term self-rated health (20). Thus far, explorations of the associations between VoA and objective health markers have been limited to physical health outcomes such as biomarkers for inflammation (21) and mortality (22). This existing literature, taken alongside established theoretical models of VoA (16, 17), would suggest that various existing physical health conditions may be associated with VoA. However, less research focuses on the connections between VoA and specific physical health conditions.

Relatedly, one in four people experiences a mental health problem of some kind each year in England (23) and about 10.7% of the global population has a mental health condition (24). People with a mental health condition may be at a greater risk for experiences that may make them observe for age-related losses (25). Considering depression, for example, 20-year increase in self-reported depression was found related to more negative VoA (20). Further, a recent study compared levels of perceived age-related losses among four groups: (1) people with current and past clinical depression, (2) people with current but not past clinical depression, (3) people with past but not current clinical depression, and (4) people with neither past nor current clinical depression (26). People with both past and current clinical depression reported more negative VoA than the other groups (26).

Unfortunately, the existing scholarship on VoA and mental health conditions is largely limited to depression and anxiety. However, older adults frequently have other mental health conditions. For example, an estimated 11–15% of community-dwelling older adults have a personality disorder (27). Thus, broadening the literature on the connections between VoA and various mental health conditions is urgent.

Only a few studies have simultaneously explored how various physical and mental health conditions were differentially associated with VoA. Schönstein et al. (28) analyzed how physical and mental health conditions at baseline predicted VoA at follow-up (median of 7.7 years) in a sample of 526 German middle-aged and older adults (28). The authors analyzed the VoA constructs of subjective age (how old people feel relative to their chronological age) and attitudes toward own aging (how people self-appraise their aging). They found that, when including a range of both physical and mental health conditions in a single statistical model, back pain, hypertension, depression, anxiety, and rheumatism were most strongly related to more negative attitudes toward own aging, and rheumatism, anxiety, and cancer were most strongly related to participants feeling older than their chronological age. The use of both subjective age and attitudes toward one’s own aging are useful, but additional studies that explore other VoA constructs are necessary. Indeed, the measures used to assess VoA in Schönstein et al.’s study (28) provide a simplified assessment of people’s VoA as they position the individual on a continuum with positive VoA on one hand and negative VoA on the other hand. This is a limitation as people can have co-existing positive and negative VoA. Moreover, as compared to positive VoA, negative VoA are typically more strongly associated with health outcomes (29). Health conditions may also be differentially related to positive and negative VoA. Thus, utilizing a multi-dimensional measure of VoA would be helpful to further understand how various health conditions are associated with VoA.

### The value of awareness of age-related gains and losses

The Awareness of Age-Related Change (AARC) construct captures both the positive (AARC-gains) and negative (AARC-losses) perceptions adults have of the changes they may observe in their daily life and attribute to aging (16, 30). In line with developmental theories and evidence (31, 32), AARC assumes that experiences and perceptions of gains and losses can coexist in five life and behavioral domains: cognitive functioning, social-emotional and social-cognitive functioning, health and physical functioning, interpersonal relations, and lifestyle and engagement. Moreover, the AARC conceptual framework assumes that physical and mental health influence levels of AARC-gains and AARC-losses (16, 30). A qualitative study asked people aged 70–88 years to document on a daily basis whether they have had any positive or negative age-related experience across the AARC life domains (17). This study found that 70% of participants reported age-related experiences in their physical health and, out of these, 82.6% reported negative age-related experiences including the experience of illnesses and health conditions. Such a finding highlights how frequently people recognize age-related changes on a daily basis, and how often these changes are bound to health conditions and are thus attributed as negative.

Quantitative research has thus far mainly focused on the associations of lower AARC-gains and higher AARC-losses with poorer cross-sectional and future scores on subjective indicators of physical health, such as functional status, pain, sleep, and physical symptoms (29, 33–40), and mental health, such as depression (9, 29). Only two studies have investigated more granular associations between various types of both physical and mental health conditions and AARC-gains and AARC-losses. Dunsmore and Neupert’s (41) analysis focused on arthritis, finding that people with arthritis had higher AARC-losses, but not lower AARC-gains, compared to individuals without arthritis. Rupprecht and colleagues (42) analyzed the associations between AARC-gains and AARC-losses and six conditions that were diagnosed in the previous 2 years. However, people who have had a health condition for longer than 2 years may be those who are objectively experiencing, and consequently perceiving, more age-related changes. It is therefore important to complement the analyses conducted by Rupprecht et al. (42) through exploration of associations between health conditions and AARC, regardless of when participants received the diagnosis.

### The present study

We investigated, in a large sample of people aged 50+, the cross-sectional associations between 26 types of lifetime physical and mental health conditions with AARC-gains and AARC-losses. First, informed by previous evidence suggesting that health is more closely connected to AARC-losses than AARC-gains (26, 41, 42), we hypothesized that a higher number of physical and mental health conditions would be related to higher AARC-losses but not to AARC-gains. Further, we hypothesized that the interaction between AARC-losses and health conditions would interact with age, with older participants having a stronger association between the number of health conditions and AARC-losses.

Regarding the type of health conditions, we expected stronger associations with AARC-losses for those health conditions that are more typically socially ascribed to old age (e.g., arthritis, osteoporosis, mild cognitive impairment, cardiovascular disease, and Parkinson’s disease) compared to those conditions that are less associated with old age (e.g., asthma and epilepsy). Lastly, due to ruminative thinking, we expect that those with depressive and anxiety symptoms are most likely to pay attention to, and therefore report, age-related losses, compared to people with other mental health conditions (9, 26).

## Materials and methods

### Study design and protocol

This study uses 2020 secondary data from the UK PROTECT cohort (Platform for Research Online to investigate Genetics and Cognition in Ageing). UK PROTECT inclusion criteria were being a UK resident, English speaker, aged 50+, having access to the internet, and lack of self-reported diagnosis of dementia at baseline (which started in 2015). During recruitment the UK PROTECT study was publicized by national advertisements that directed potential participants to the PROTECT study website where they could enroll to the study. Individuals aged 50+ from existing research cohorts (including Exeter 10,000, Join Dementia Research, and Brains for Dementia Research) were also invited to take part to the study via email. When joining the study participants provided informed consent online. UK PROTECT has ethical approval from the London Bridge NHS Research Ethics Committee and Health Research Authority (Ref: 13/LO/1578). For the scope of this study, in 2020 UK PROTECT participants were optionally invited to complete the AARC questionnaire; 5,353 participants did so. This study comprises those 3,786 participants who, in addition to completing the AARC questionnaire, also self-reported, in 2020, whether they had diagnoses of physical and mental health conditions. Descriptive statistics for those who provided data on AARC and health conditions and those who did not are in Supplementary Table S1.

### Measures

#### Socio-demographics

Sociodemographic variables comprised age, sex, education (*secondary education; post-secondary education; vocational qualifications; undergraduate degrees; post-graduate degrees; doctorates*), martial status (*married/co-habiting/in a civic partnership* vs. *never married/widowed/divorced*), employment status (*employed* vs. not *employed*), and ethnicity.

#### Awareness of age-related change

We measured VoA by capturing participants AARC with a 10-item questionnaire [AARC-10 SF; (43)] comprising five items assessing AARC-gains and five items assessing AARC-losses. An item in each of the AARC-gains and AARC-losses subscales assesses one of the five AARC life and behavioral domains. Questionnaire items are based on the positive and negative age-related changes US and German adults aged 40+ reported noticing in their daily life during focus groups, interviews, and diaries. AARC items capture the changes adults may perceive in their lives, irrespective of whether these match objective changes.

Each item starts with the stem: “*With my increasing age, I realize that…*.” Respondents rate how much items apply to them (1 = *not at all*; 2 = *a little bit*; 3 = *moderately*; 4 = *quite a bit*; 5 = *very much*).

**Table:** 

Domain	AARC-gains	AARC-losses
Cognitive functioning	“…*I have more experience and knowledge to evaluate things and people*”	“…*My mental capacity is declining*”
Social-emotional and social cognitive functioning	“…*I have a better sense of what is important for me*”	“…*I find it harder to motivate myself*”
Health and physical functioning	*“…I pay more attention to my health”*	*“…I have less energy”*
Interpersonal relations	“…*I appreciate relationships and people much more*”	“…*I feel more dependent on the help of others*”
Lifestyle and engagement	“…*I have more freedom to live my days the way I want*”	“…*I have to limit my activities*”

Scores can be obtained for the AARC-gains and AARC-losses subscales by summing the five items within the respective subscales. Higher scores indicate higher AARC-gains/losses (range: 5–25). In this sample Cronbach’s alpha for internal consistency for the AARC-gains subscale was 0.74 and for the AARC-losses subscale was 0.81, indicating good scales reliability.

#### Physical health conditions

Participants indicated whether they received (1 = yes, 0 = no) at any point in their life a diagnosis of each of the 19 examined health conditions (reported in Table 1). Number of physical health conditions was a count of the conditions reported. Although cancer in full remission is not a current physical health condition, we kept it in the count as past history of cancer may influence VoA, our study outcome (44).

**Table 1:** Hierarchical regression models with sociodemographic variables, number of physical and mental health conditions, and their interactions with age as predictors of AARC-gains and AARC-losses.

Predictors	Beta (95% CI); *p*-value	Standardized beta
**Hierarchical regression models with sociodemographic variables and number of physical and mental health conditions as predictors of AARC-gains**		
Age	−0.02 (−0.05; 0.01); 0.242	−0.03
Sex	1.28 (0.98; 1.59); <0.001	0.14
Education	−0.10 (−0.19; −0.01); 0.029	−0.04
Marital status	−0.27 (−0.59; 0.05); 0.094	−0.03
Working status	−0.19 (−0.49; 0.12); 0.230	−0.02
Number of physical health conditions	0.36 (−0.60; 1.32); 0.462	0.12
Number of physical health conditions X age	−0.004 (−0.02; 0.01), 0.606	−0.09
Number of mental health conditions	−0.14 (−1.91; 1.63); 0.879	−0.03
Number of mental health conditions X age	0.002 (−0.02; 0.03); 0.862	0.03
**Hierarchical regression models with sociodemographic variables and number of physical and mental health conditions as predictors of AARC-losses**		
Age	0.10 (0.07; 0.12); <0.001	0.20
Sex	−0.47 (−0.73; −0.22); <0.001	−0.06
Education	−0.12 (−0.20; −0.04); 0.002	−0.05
Marital status	−0.30 (−0.57; −0.02); 0.027	−0.04
Working status	−0.13 (−0.38; 0.13); 0.336	−0.02
Number of physical health conditions	0.38 (−0.42; 1.18); 0.351	0.14
Number of physical health conditions X age	0.003 (−0.01; 0.01); 0.598	0.08
Number of mental health conditions	2.74 (1.26; 4.21); <0.001	0.60
Number of mental health conditions X age	−0.03 (−0.05; −0.01); 0.007	−0.44

#### Mental health conditions

Participants indicated whether they received (1 = yes; 0 = no) at any point in their life a diagnosis of each of the 7 examined mental health conditions. Number of mental health conditions was a count of the diagnoses reported.

### Study analyses

We used hierarchical linear regression models to explore, at cross-sectional level, the additional explanatory value of number of physical and mental health conditions (predictor), in addition to socio-demographic variables, on AARC-gains and AARC-losses. Model 1 included only socio-demographic variables as predictors. Model 2 added number of physical health conditions as predictor. Model 3 added number of mental health conditions as predictor. We also tested a fourth model adding the interactions of number of physical and mental health conditions with age as additional predictors. We also used linear regression models to explore the cross-sectional associations of presence of each of the examined physical and mental health conditions (predictors) with AARC-gains and AARC-losses. In a further step, we used multiple hierarchical linear regression models to explore the predictive value of each physical and mental health condition on AARC-gains and AARC-losses above and beyond the effect of other conditions. To quantify the associations we reported standardized regression coefficients (β; effects sizes). Values ≤0.09 were considered negligible, 0.10–0.29 small, 0.30–0.49 moderate, and ≥0.50 large (45). We conducted all analyses using STATA version 17 (46).

## Results

### Descriptive statistics

The sample comprised 3,786 adults between the ages of 52 and 96 (mean age = 67.04 years, SD = 6.88 years; Table 2). The majority of the sample was women (78.0%). Educational achievements varied. The majority were married (79.8%), and the majority were not employed (61.3%). Almost all participants were of White ethnicity (98.7). Participants reported moderate age-related gains (*M* = 18.31; SD = 3.74) and few age-related losses (*M* = 10.14; SD = 3.36). 68.8% of the sample had at least one physical health condition, and 25.6% had at least one mental health condition.

**Table 2:** Descriptive statistics for the overall study sample.

	Study sample (*n* = 3,786)
Age; *M* (SD)	67.04 (6.88)
Women, *n* (%)	2,953 (78.0)
**Education, *n* (%)**	
Secondary education	512 (13.5)
Post-secondary education	438 (11.6)
Vocational qualification	752 (19.9)
Undergraduate degree	1,309 (34.6)
Post-graduate degree	637 (16.8)
Doctorate	139 (3.7)
Married/co-habiting/in a civic partnership, *n* (%)	2,829 (79.8)
Employed, *n* (%)	1,434 (38.7)
White ethnicity, *n* (%)	3,737 (98.7)
Awareness of age-related gains, *M* (SD)	18.31 (3.74)
Awareness of age-related losses, *M* (SD)	10.14 (3.36)
Number of physical health conditions, M (SD)	1.32 (1.26)
**Physical health conditions, *n* (%)**	
Zero	1,182 (31.2)
One	1,170 (30.9)
Two	806 (21.3)
Three	407 (10.8)
Four	150 (4.0)
Five	50 (1.3)
Six	15 (0.4)
Seven	4 (0.1)
Eight	2 (0.1)
Number of mental health conditions, *M* (SD)	0.38 (0.76)
**Mental health conditions, *n* (%)**	
Zero	2,816 (74.4)
One	652 (17.2)
Two	210 (5.6)
Three	82 (2.2)
Four	20 (0.5)
Five	4 (0.1)
Six	2 (0.1)

### Number of physical and mental health conditions

After having included age, sex, education, marital status and employment status as cross-sectional correlates of AARC-gains and AARC-losses (Model 1; Table 3), a higher number of physical health conditions was associated with higher AARC-gains and higher AARC-losses (Model 2). A higher number of mental health conditions was associated with higher AARC-losses, but not to lower AARC-gains (Model 3). After having included in the model the interactions between number of physical and mental health conditions with age, number of physical health conditions and its interaction with age, as well as number of mental health conditions and its interaction with age, were no longer significantly associated with AARC-losses. After having included in the model the interactions between physical and mental health conditions with age, number of physical health conditions and its interaction with age were also not significant predictor of AARC-gains. Higher number of mental health conditions were instead associated with higher AARC-gains, especially among those who were older (Model 4; Table 1). All models explained 3% of the variance in AARC-gains. Models 1, 2, 3, and 4 explained 8, 14, 16, and 16% of the variance in AARC-losses, respectively. The R2 changes from model 1 to model 2 and from model 2 to model 3 were significant.

**Table 3:** Hierarchical regression models with sociodemographic variables and number of physical and mental health conditions as predictors of AARC-gains and AARC-losses.

Predictors	Model 1		Model 2		Model 3	
	Beta (95% CI); *p*-value	Standardized beta	Beta (95% CI); *p*-value	Standardized beta	Beta (95% CI); *p*-value	Standardized beta
**Hierarchical regression models with sociodemographic variables and number of physical and mental health conditions as predictors of AARC-gains**						
Age	−0.02 (−0.04; 0.004); 0.115	−0.03	−0.02 (−0.05; −0.003)	−0.04	−0.02 (−0.05; −0.0001); 0.049	−0.04
Sex	1.29 (0.98;1.59); <0.001	0.14	1.29 (98; 1.59); <0.001	0.14	1.28 (0.98; 1.59); <0.001	0.14
Education	−0.10 (−0.20; −0.01); 0.024	−0.04	−0.10 (−0.19; −0.01); 0.020	−0.04	−0.10 (−0.19; −0.01); 0.029	−0.04
Marital status	−0.29 (−0.62; 0.03); 0.073	−0.03	−0.27 (−0.59; 0.05); 0.095	−0.03	−0.27 (−0.59; 0.05); 0.097	−0.03
Working status	−0.20 (−0.50; 0.11); 0.202	−0.03	−0.20 (−0.50; 0.11); 0.214	−0.03	−0.19 (−0.50; 0.11); 0.212	−0.03
Number of physical health conditions			0.11 (0.01; 0.21); 0.034	0.04	0.11 (0.01; 0.21); 0.039	0.04
Number of mental health conditions					0.02 (−0.15; 0.19); 0.802	0.004
**Hierarchical regression models with sociodemographic variables and number of physical and mental health conditions as predictors of AARC-losses**						
Age	0.12 (0.10; 0.14); <0.001	0.24	0.08 (0.07; 0.10), <0.001	0.17	0.09 (0.07; 0.11); <0.001	0.19
Sex	−0.44 (−0.70; −0.18); 0.001	−0.06	−0.43 (−0.69; −0.18); 0.001	−0.05	−0.49 (−0.74; −0.24); <0.001	−0.06
Education	−0.14 (−0.22, 0.07); <0.001	−0.06	−0.13 (−0.20; −0.05); 0.001	−0.05	−0.12 (−0.20; −0.05); 0.002	−0.05
Marital status	−0.48 (−0.75; −0.20); 0.001	−0.06	−0.36 (−0.63; −0.09); 0.009	−0.04	−0.31 (−0.58; −0.05); 0.021	−0.04
Working status	−0.12 (−0.39; 0.14); 0.367	−0.02	−0.09 (−0.35; 0.16); 0.479	−0.01	−0.12 (−0.38; 0.13); 0.350	−0.02
Number of physical health conditions			0.65 (0.56; 0.73); <0.001	0.24	0.59 (0.51; 0.68); <0.001	0.22
Number of mental health conditions					0.71 (0.57; 0.85); <0.001	0.15

### Type of physical and mental health conditions

Associations for each health condition and AARC-gains and AARC-losses adjusted for sociodemographic variables are in Supplementary Table S2. Table 4 reports results from multiple regressions including all physical and mental health condition as cross-sectional predictors of AARC-gains and AARC-losses. Participants with current cancer reported higher AARC-gains. The remaining physical and mental health conditions were not related to AARC-gains. Diagnoses of mild cognitive impairment, Parkinson’s disease, arthritic condition, cancer in full remission, osteoporosis, depression, anxiety disorders, and personality disorder were associated with higher AARC-losses. Among these conditions, those most strongly related to AARC-losses were depression and arthritic condition.

**Table 4:** All physical and mental health conditions included in the same multiple regression model as predictors of AARC-gains and AARC-losses.

Type of physical health condition/predictors	AARC-gains	AARC-losses
	Standardized beta (95% CI); *p*-value	
Cardiovascular disease	0.03 (−0.01; 0.07)	0.10 (0.06; 0.13)***
Diabetes	0.01 (−0.03; 0.04)	0.02 (−0.01; 0.06)
Mild cognitive impairment	0.02 (−0.01; 0.06)	0.06 (0.03; 0.09)**
Parkinson’s disease	0.02 (−0.02; 0.05)	0.06 (0.03; 0.09)**
Hypothyroidism	0.01 (−0.03; 0.04)	0.02 (−0.01; 0.05)
Hyperthyroidism	0.02 (−0.01; 0.06)	0.01 (−0.02; 0.05)
Arthritic condition	0.01 (−0.03; 0.04)	0.12 (0.09; 0.16)***
Current cancer	0.04 (0.003; 0.07)*	−0.02 (−0.05; 0.01)
Cancer in full remission	−0.01 (−0.05; 0.03)	0.05 (0.02; 0.08)**
Osteoporosis	−0.03 (−0.07; 0.003)	0.06 (0.03; 0.09)**
Asthma	0.01 (−0.03; 0.04)	0.03 (−0.003; 0.06)
Epilepsy	−0.01 (−0.05; 0.02)	−0.01 (−0.05; 0.02)
Multiple sclerosis	0.01 (−0.03; 0.04)	0.02 (−0.01; 0.05)
Depression	0.001 (−0.04; 0.04)	0.14 (0.11; 0.18)
Mania	0.01 (−0.03; 0.04)	0.01 (−0.03; 0.04)
Anxiety disorders	0.02 (−0.02; 0.06)	0.06 (0.03; 0.09)**
Eating disorder	−0.01 (−0.05; 0.02)	−0.01 (−0.05; 0.02)
Psychotic disorders	0.02 (−0.02; 0.06)	0.02 (−0.01; 0.05)
Personality disorder	−0.03 (−0.07; 0.01)	0.07 (0.04; 0.11)***

For those mental health conditions endorsed by less than 10 people we did not run linear regression models. The models are adjusted for age, sex, education, marital status, and working status. AARC-gains, Awareness of positive age-related changes; AARC-losses, Awareness of negative age-related changes; CI, Confidence Interval. Cancer in full remission means no evidence of disease. Psychotic disorders include schizophrenia. *p < 0.05. **p < 0.01. ***p < 0.001.

## Discussion

By analyzing connections between VoA and health conditions, researchers can identify who may be at a greater risk for poorer subjective aging experiences. Given the saliency of VoA to healthy aging processes, and the high prevalence of (multiple) health conditions among middle-aged and older adults, doing so is urgent. We therefore investigated the cross-sectional associations between number and type of physical and mental health conditions and VoA with the multi-dimensional Awareness of Age-Related Change (AARC) gains and losses construct.

### Key findings

#### Health conditions are associated with higher losses, but no different gains

The value of the AARC construct and its associated validated measurement tools is that they allow researchers to simultaneously explore positive vs. negative VoA. Indeed, existing research suggests that positive and negative VoA are two separate constructs rather than two extremes of a single construct (47); for example, just because someone has high negative VoA does not mean that they also have low positive VoA. The AARC construct and measure were designed to simultaneously gage positive and negative VoA across five life domains. Our analysis of how various health conditions are differentially/uniquely associated with AARC-gains vs. AARC-losses provides an initial understanding of the relationship between illness and VoA. This was in line with our hypothesis. However, against our hypothesis a greater number of physical health conditions showed a small association with higher AARC-gains. Moreover, as hypothesized, a greater total number of mental health conditions greatly impacted higher AARC-losses, especially among older people, and this effect hold true the interaction between number of mental health conditions and age. However, a greater number of mental health conditions was not associated with AARC-gains.

Further, having each of the following conditions was associated with higher AARC-losses, but no different AARC-gains: mild cognitive impairment, Parkinson’s disease, arthritis, osteoporosis, cancer in remission, depression, anxiety, a psychotic disorder, and a personality disorder. These results are in line with our hypothesis and extend to additional health conditions existing scholarship which suggests that AARC-losses are more connected to health than AARC-gains (41, 42). More specifically we identified which specific conditions should be further investigated as potentially related to negative VoA in midlife and later life.

It is important to note that the small positive association between physical health conditions and AARC-gains may be a sign of positive coping with illness and resilience. However, further longitudinal investigation is warranted to explore whether AARC-gains match longer term objective gains in these groups, such as reduced functional decline and mortality, as well as whether it is related to greater resilience and positive coping.

##### Cancer may be unique

Current cancer was the lone health condition that was associated with AARC-gains. Having cancer at the time of the survey was associated with slightly higher AARC-gains, but no different AARC-losses. Though we are unaware of the type of cancer and the life-expectancy prognosis in this sample, cancer can be and is often perceived as a terminal diagnosis. The terminal nature of cancer contrasts with many of the other health conditions in our sample, which are mostly manageable chronic conditions. Cancer’s association with AARC-gains may be a reflection of the way the AARC measure we utilized in this study captured AARC-gains. It is plausible that AARC-gains items such as “*I have a better sense of what is important for me*” and “*I appreciate relationships and people much more*” would be endorsed more readily by someone facing a potentially terminal illness because people who sense death is closer are more likely to prioritize close, meaningful relationships than other goals (48). Overall, that cancer was associated with AARC-gains in a way that was unlike every other health condition we analyzed highlights the unique ways in which certain health conditions may differentially be related to positive vs. negative VoA.

#### Health conditions that more frequently occur in later life are more closely linked to age-related losses than other health conditions

We hypothesized that age would be a significant moderator for the associations between both physical and mental health conditions and AARC-losses. However, age was only a significant moderator of the association between the number of mental health conditions and AARC-losses. That is, the older a person in our sample was, the stronger the connection between more mental health conditions and AARC-losses. Age was not associated with the association between the number of physical health conditions and AARC-losses. However, many type of physical health conditions were associated with AARC-losses, suggesting it may be more about the type of physical health condition rather than the number of total conditions.

As we hypothesized, among all the physical health conditions we investigated, those related to more AARC-losses were an arthritic condition, osteoporosis, mild cognitive impairment, and Parkinson’s disease. These conditions become more frequent with increasing age (49), and may be socially ascribed to older age. Thus, they may be highly salient markers of age-related losses and therefore more connected to AARC-losses than other conditions that are not as closely aligned with older age (i.e., diabetes or asthma).

Arthritis and osteoporosis are associated with persistent pain that can impact a person’s ability to independently engage in daily tasks (50, 51) and hence be salient in one’s evaluation of age-related losses. Indeed, the associations of pain and functional limitations (29) with AARC-losses have been established (52). Pain is also highly associated with negative emotions (53), which could make someone more prone to feel more negatively about their own aging. It is important to note that in our sample multiple sclerosis, a health condition with pain symptoms, but not strongly associated with age, was not associated with either AARC-gains or AARC-losses. That pain-related conditions that are typically associated with later life (arthritis and osteoarthritis) were associated with AARC-losses but a pain-related condition that is not necessarily associated with later life (multiple sclerosis) was not could underscore the importance of a health condition being social ascribed as one that happens in later life.

#### In addition to depression and anxiety, personality disorders are associated with AARC

In line with existing studies depression was associated with AARC-losses (1, 9, 29). There are several possible explanations for the relationship between depression and AARC-losses. Each of these possibilities offers an avenue for future research. First, it may be that people with a history of depression are at higher risk of experiencing cognitive difficulties, which can be a direct consequence of depression (54, 55). Relatedly, depression can be a prodrome and accelerating factor for pathological cognitive decline (56). Also, depressive symptomatology can be present among those who face particularly challenging physical health conditions such as cardiovascular and cerebrovascular diseases (56, 57). Finally, people with depression may also be more prone to reflect (i.e., ruminate) over the objective age-related losses they may experience (9). For instance, generally people with negative stable global views about themselves and the word may be both more prone to depressive symptoms and to more promptly recognize the losses they experience and to repetitively think over them without taking action (58–60).

Although there is a plethora of literature linking personality to VoA (61), our study found for the first time that more AARC-losses are related to personality disorders. This may be due to the more restricted social life that individuals with personality disorders typically have (62–66). Poorer social engagement and social support are indeed related to more negative VoA (67–74). The number of participants in our sample with a personality disorder was very small (*n* = 11), therefore this finding should be taken as suggestive. Nonetheless, it encourages the exploration of other mental health conditions beyond depression and anxiety as correlates of VoA. Extending this existing scholarship to study personality disorders would be valuable.

### Limitations

This study has important limitations. First, though establishing cross-sectional associations between AARC and physical and mental health conditions was an important first step, analyzing these associations longitudinally is necessary. Only cross-sectional data were available to us at the time of analysis. However, the ongoing nature of the UK PROTECT study will make it possible to explore this in future studies. Second, participants did not report how long they had each diagnosis, when they were diagnosed, or severity of conditions. Therefore, we were unable to fully compare health conditions that were long-standing (or even life-long) to those that were more recently diagnosed. Further, though most of the health conditions we analyzed are chronic conditions that, once diagnosed, people have to manage their entire lives, some conditions may be cured or reversed (e.g., diabetes). As such, participants may have had some conditions in the past but not at the time of the survey. Because health conditions can contribute to cumulative health disadvantages over a lifetime, it will be important for future research to consider the duration of each diagnosis. Severity of the health conditions should also be considered as a variable that may moderate associations between AARC and health conditions.

Third, for some of the investigated conditions the number of participants reporting the given condition was very low. In some instances, small sample size impeded our ability to estimate an association; in other cases, we computed the estimation but results should be taken as suggestive. Fourth, participants were excluded from study analyses based on self-reported diagnosis of dementia. This is a limitation compared to the use of objective indicators such as doctor-reported diagnosis of dementia. Fifth, though we controlled for gender in our analyses, our sample was mostly comprised of women. A sample with greater gender diversity would allow for more robust gender comparisons given that gender plays a role in VoA (75) and that certain health conditions are more common in older women than men (e.g., osteoporosis) (76). Nonetheless this study included a variety of health conditions and an age-diverse sample. Moreover, it provides initial evidence for the ways in which specific health conditions are connected to AARC, and should encourage future research on specific health conditions.

## Conclusion

A higher number of physical and mental health illnesses are associated with higher levels of awareness of age-related losses. Specific health conditions, such as arthritis, depression, and personality disorder may be related to greater vulnerability to negative age-related experiences. These individuals could benefit from some sort of intervention that helps them accept and/or adjust to health-related losses.

## Data availability statement

The original contributions presented in the study are included in the article/Supplementary material, further inquiries can be directed to the corresponding author.

## Ethics statement

The studies involving human participants were reviewed and approved by The UK PROTECT study, from which data was used, has ethical approval from the London Bridge NHS Research Ethics Committee and Health Research Authority (Ref: 13/LO/1578). The patients/participants provided their written informed consent to participate in this study.

## Author contributions

SS and ST equally designed the Current study, wrote the manuscript, developed the research questions, and drafted the manuscript. SS conducted study analyses. HB, CB, AC, and AH contributed to collection and acquisition of UK PROTECT data and provided feedback and assistance with editing of the manuscript. All authors contributed to the article and approved the submitted version.

## References

[1] Dutt AJ Gabrian M Wahl H-W. Awareness of age-related change and depressive symptoms in middle and late adulthood: longitudinal associations and the role of self-regulation and calendar age. J Gerontol B Psychol Sci Soc Sci. (2016) 73:gbw095–53. doi: 10.1093/geronb/gbw095 27534425

[2] Levy BR Myers LM. Preventive health behaviors influenced by self-perceptions of aging. Prev Med. (2004) 39:625–9. doi: 10.1016/j.ypmed.2004.02.029, PMID: 15313104

[3] Wurm S Tomasik MJ Tesch-Römer C. On the importance of a positive view on ageing for physical exercise among middle-aged and older adults: cross-sectional and longitudinal findings. Psychol Health. (2010) 25:25–42. doi: 10.1080/08870440802311314, PMID: 20391205

[4] Beyer AK Wolff JK Warner LM Schuz B Wurm S. The role of physical activity in the relationship between self-perceptions of ageing and self-rated health in older adults. Psychol Health. (2015) 30:671–85. doi: 10.1080/08870446.2015.1014370, PMID: 25720739

[5] Wurm S Warner LM Ziegelmann JP Wolff JK Schuz B. How do negative self-perceptions of aging become a self-fulfilling prophecy? Psychol Aging. (2013) 28:1088–97. doi: 10.1037/a0032845, PMID: 24128074

[6] Beyer A-K Wiest M Wurm S. There is still time to be active: self-perceptions of aging, physical activity, and the role of perceived residual lifetime among older adults. J Aging Phys Act. (2019) 27:807–15. doi: 10.1123/japa.2018-0380 30859900

[7] Hooker K Mejía ST Phibbs S Tan EJ Stevens J. Effects of age discrimination on self-perceptions of aging and cancer risk behaviors. The Gerontologist. (2019) 59:S28–37. doi: 10.1093/geront/gny183 31100138

[8] Brothers AF Kornadt AE Nehrkorn-Bailey A Wahl H-W Diehl MK. The effects of age stereotypes on physical and mental health are mediated by self-perceptions of aging. J Gerontol B Psychol Sci Soc Sci. (2020) 76:845–57. doi: 10.1093/geronb/gbaa176 PMC8063677 33057726

[9] Dutt AJ Wahl H-W Rupprecht F. Mindful vs. mind full: processing strategies moderate the association between subjective aging experiences and depressive symptoms. Psychol Aging. (2018) 33:630–42. doi: 10.1037/pag0000245, PMID: 29708386

[10] Seidler AL Wolff JK. Bidirectional associations between self-perceptions of aging and processing speed across 3 years. J Gerontopsychol Geriatr Psychiatry. (2017) 30:49–59. doi: 10.1024/1662-9647/a000165

[11] Kaspar R Wahl H-W Diehl MK. Awareness of age-related change as a behavioral determinant of survival time in very old age. Front Psychol. (2021) 12:727560. doi: 10.3389/fpsyg.2021.727560, PMID: 34650486 PMC8505719

[12] Robertson DA Savva GM King-Kallimanis BL Kenny RA. Negative perceptions of aging and decline in walking speed: a self-fulfilling prophecy. PLoS One. (2015) 10:e0123260. doi: 10.1371/journal.pone.0123260 25923334 PMC4414532

[13] Stephan Y Sutin AR Luchetti M Terracciano A. Feeling older and the development of cognitive impairment and dementia. J Gerontol B Psychol Sci Soc Sci. (2017) 72:966–73. doi: 10.1093/geronb/gbw085 27436103 PMC5927095

[14] Stephan Y Sutin AR Luchetti M. Terracciano a. the prospective relationship between subjective aging and inflammation: evidence from the health and retirement study. Psychophysiology. (2022) 60:e14177. doi: 10.1111/psyp.14177 36124383

[15] Knight RL Chalabaev A McNarry MA Mackintosh KA Hudson J. Do age stereotype-based interventions affect health-related outcomes in older adults? A systematic review and future directions. Br J Health Psychol. (2021) 27:338–73. doi: 10.1111/bjhp.12548, PMID: 34254707

[16] Diehl MK Wahl H-W. Awareness of age-related change: examination of a (mostly) unexplored concept. J Gerontol B Psychol Sci Soc Sci. (2010) 65B:340–50. doi: 10.1093/geronb/gbp110 20008026 PMC2853600

[17] Miche M Wahl H-W Diehl MK Oswald F Kaspar R Kolb M. Natural occurrence of subjective aging experiences in community-dwelling older adults. J Gerontol B Psychol Sci Soc Sci. (2014) 69:174–87. doi: 10.1093/geronb/gbs164, PMID: 23325506 PMC12124237

[18] Public Health England. Chapter 1: population change and trends in life expectancy. (2018). Available at: https://www.gov.uk/government/publications/health-profile-for-england-2018/chapter-1-population-change-and-trends-in-life-expectancy.

[19] AgeUK. Briefing: Health and Care of Older People in England 2019. (2019). Available at: https://www.ageuk.org.uk/globalassets/age-uk/documents/reports-and-publications/reports-and-briefings/health--wellbeing/age_uk_briefing_state_of_health_and_care_of_older_people_july2019.pdf

[20] Sabatini S Siebert JS Diehl MK Brothers A Wahl H-W. Identifying predictors of self-perceptions of aging based on a range of cognitive, physical, and mental health indicators: twenty-year longitudinal findings from the ILSE study. Psychol Aging. (2022) 37:486–502. doi: 10.1037/pag0000668 34941356 PMC10413976

[21] Stephan Y Sutin AR Terracciano A. Younger subjective age is associated with lower C-reactive protein among older adults. Brain Behav Immun. (2015) 43:33–6. doi: 10.1016/j.bbi.2014.07.019, PMID: 25108213

[22] Stephan Y Sutin AR Terracciano A. Subjective age and mortality in three longitudinal samples. Psychosom Med. (2018) 80:659–64. doi: 10.1097/PSY.0000000000000613, PMID: 29864106 PMC6345273

[23] Mental Health Foundation. Available at: https://www.mentalhealth.org.uk/statistics/mental-health-statistics-older-people (Accessed June 30, 2023).

[24] Our world in data. (2023) Available at: https://ourworldindata.org/mental-health (Accessed June 30, 2023).

[25] Zisook S Lesser I Stewart JW Wisniewski SR Balasubramani GK Fava M. Effect of age at onset on the course of major depressive disorder. Am J Psychiatr. (2007) 164:1539–46. doi: 10.1176/appi.ajp.2007.06101757 17898345

[26] Sabatini S Dritschel B Rupprecht FS Ukoumunne OC Ballard C Brooker H. Rumination moderates the longitudinal associations of awareness of age-related change with depressive and anxiety symptoms. Aging Ment Health. (2023) 1-9:1–9. doi: 10.1080/13607863.2023.2176820 36762688

[27] Penders KAP Peeters IGP Metsemakers JFM van Alphen SPJ. Personality disorders in older adults: a review of epidemiology, assessment, and treatment. Curr Psychiatry Rep. (2020) 22:14. doi: 10.1007/s11920-020-1133-x, PMID: 32025914 PMC7002365

[28] Schönstein A Dallmeier D Denkinger M Rothenbacher D Klenk J Bahrmann A. Health and subjective views on aging: longitudinal findings from the ActiFE Ulm study. J Gerontol Ser B. (2021) 76:1349–59. doi: 10.1093/geronb/gbab023, PMID: 33528511 PMC8363042

[29] Sabatini S Silarova B Martyr A Collins R Ballard C Anstey KJ. Associations of awareness of age-related change with emotional and physical well-being: a systematic review and meta-analysis. The Gerontologist. (2020) 60:e477–90. doi: 10.1093/geront/gnz101 31350849 PMC7427487

[30] Diehl MK Wahl H-W Barrett AE Brothers AF Miche M Montepare JM. Awareness of aging: theoretical considerations on an emerging concept. Dev Rev. (2014) 34:93–113. doi: 10.1016/j.dr.2014.01.001, PMID: 24958998 PMC4064469

[31] Baltes PB. Theoretical propositions of life-span developmental psychology: on the dynamics between growth and decline. Dev Psychol. (1987) 23:611–26. doi: 10.1037/0012-1649.23.5.611

[32] Heckhausen J Dixon RA Baltes PB. Gains and losses in development throughout adulthood as perceived by different adult age groups. Dev Psychol. (1989) 25:109–21. doi: 10.1037/0012-1649.25.1.109

[33] Sabatini S Ukoumunne OC Ballard C Brothers AF Kaspar R Collins R. International relevance of two measures of awareness of age-related change (AARC). BMC Geriatr. (2020) 20:359. doi: 10.1186/s12877-020-01767-6, PMID: 32957978 PMC7507664

[34] Neri AL Wahl H-W Kaspar R Diehl MK Batistoni SST Cachioni M. Psychometric study of the awareness of age-related change (AARC) short scale translated to Portuguese, applied to Brazilian older adults. Demen Neuropsychol. (2021) 15:230–8. doi: 10.1590/1980-57642021dn15-020011, PMID: 34345365 PMC8283873

[35] Sabatini S Ukoumunne OC Brothers A Diehl MK Wahl H-W Ballard C. Differences in awareness of positive and negative age-related changes accounting for variability in health outcomes. Eur J Ageing. (2022) 19:1087–97. doi: 10.1007/s10433-021-00673-z, PMID: 36506661 PMC9729481

[36] Wilton-Harding B Windsor TD. Awareness of age-related change, future time perspective, and implications for goal adjustment in older adulthood. Aging Ment Health. (2021) 26:1189–97. doi: 10.1080/13607863.2021.1893269, PMID: 33682540

[37] Sabatini S Ukoumunne OC Ballard C Collins R Corbett A Brooker H. Cross-sectional and longitudinal associations between subjective sleep difficulties and self-perceptions of aging. Behav Sleep Med. (2021) 20:732–61. doi: 10.1080/15402002.2021.1994405 34689666

[38] Zhu X Neupert SD. Dynamic awareness of age-related losses predict concurrent and subsequent changes in daily inductive reasoning performance. Br J Dev Psychol. (2020) 39:282–98. doi: 10.1111/bjdp.12344, PMID: 32734643

[39] Sabatini S Ukoumunne OC Ballard C Collins R Anstey KJ Diehl MK. Cross-sectional association between objective cognitive performance and perceived age-related gains and losses in cognition. Int Psychogeriatr. (2021) 33:727–41. doi: 10.1017/S1041610221000375, PMID: 33849677

[40] Zhang S Neupert SD. Awareness of aging mediates the relationship between health and control beliefs: a micro-longitudinal study. Innov Aging. (2020) 4:448. doi: 10.1093/geroni/igaa057.1449

[41] Dunsmore VJ Neupert SD. No pain, no gain? Personality associations with awareness of aging depend on arthritis. Front Psychol. (2022) 13:863152. doi: 10.3389/fpsyg.2022.863152, PMID: 35756270 PMC9218331

[42] Rupprecht FS Sabatini S Diehl MK Gerstorf D Kaspar R Schilling OK. Awareness of age-related change in the context of major life events. Frontiers. Psychiatry. (2022) 13:13. doi: 10.3389/fpsyt.2022.954048 PMC9650375 36386972

[43] Kaspar R Gabrian M Brothers AF Wahl H-W Diehl MK. Measuring awareness of age-related change: development of a 10-item short form for use in large-scale surveys. The Gerontologist. (2019) 59:e130–40. doi: 10.1093/geront/gnx213, PMID: 29401221 PMC6524483

[44] Laryionava K Schönstein A Heußner P Hiddemann W Winkler EC Wahl H-W. Experience of time and subjective age when facing a limited lifetime: the case of older adults with advanced cancer. J Aging Health. (2022) 34:736–49. doi: 10.1177/08982643211063162, PMID: 34967672 PMC9446453

[45] Cohen J. Statistical power analysis for the behavioral sciences. Hillsdale, NJ: Lawrence Earlbaum Associates (1988).

[46] StataCorp. Stata statistical software: Release 17 College Station, TX (2021).

[47] Turner SG Hooker K Geldhof GJ. Self-perceptions of aging: factorial structure and invariance by gender. The Gerontologist. (2020) 61:425–9. doi: 10.1093/geront/gnaa059 32578845

[48] Carstensen LL Hershfield HE. Beyond stereotypes: using socioemotional selectivity theory to improve messaging to older adults. Curr Dir Psychol Sci. (2021) 30:327–34. doi: 10.1177/09637214211011468, PMID: 34366582 PMC8340497

[49] Centers for Disease Control and Prevention https://www.cdc.gov/chronicdisease/data/surveillance.htm (Accessed June 30, 2023).

[50] Ahlstrand I Björk M Thyberg I Börsbo B Falkmer T. Pain and daily activities in rheumatoid arthritis. Disabil Rehabil. (2012) 34:1245–53. doi: 10.3109/09638288.2011.638034 22191990

[51] Hunter DJ Riordan EA. The impact of arthritis on pain and quality of life: an a ustralian survey. Int J Rheum Dis. (2014) 17:149–55. doi: 10.1111/1756-185X.12232, PMID: 24267825

[52] Sabatini S Ukoumunne OC Ballard C Collins R Corbett A Brooker H. The cross-sectional relationship between pain and awareness of age-related changes. Br J Pain. (2020) 15:335–44. doi: 10.1177/2049463720961798 34377459 PMC8339953

[53] Lerman SF Rudich Z Brill S Shalev H Shahar G. Longitudinal associations between depression, anxiety, pain, and pain-related disability in chronic pain patients. Psychosom Med. (2015) 77:333–41. doi: 10.1097/PSY.0000000000000158, PMID: 25849129

[54] Bunce D Batterham PJ Christensen H Mackinnon AJ. Causal associations between depression symptoms and cognition in a community-based cohort of older adults. Am J Geriatr Psychiatry. (2014) 22:1583–91. doi: 10.1016/j.jagp.2014.01.004, PMID: 24502823

[55] Bhalla RK Butters MA Becker JT Houck PR Snitz BE Lopez OL. Patterns of mild cognitive impairment after treatment of depression in the elderly. Am J Geriatr Psychiatry. (2009) 17:308–16. doi: 10.1097/JGP.0b013e318190b8d8, PMID: 19307859 PMC2782929

[56] Piras F Banaj N Porcari DE Spalletta G. Later life depression as risk factor for developing dementia: epidemiological evidence, predictive models, preventive strategies and future trends. Minerva Med. (2021) 112:456–66. doi: 10.23736/S0026-4806.21.07571-6, PMID: 34056888

[57] Kales HC Maixner DF Mellow AM. Cerebrovascular disease and late-life depression. Am J Geriatr Psychiatry. (2005) 13:88–98. doi: 10.1097/00019442-200502000-00002 15703317

[58] Beck AT. Cognitive models of depression In: Leathy RL Dowd ET, editors. Clinical advances in cognitive psychotherapy: Theory and application, vol. 14. New York: Springer Publishing Co (2002). 29–61.

[59] Williams M Watts FN MacLeod C Mathews A. Cognitive psychology and emotional disorders. Chichester: John Wiley & Sons (1988).

[60] Abramson LY Alloy LB Hankin BL Haeffel GJ MacCoon DG Gibb BE. Cognitive vulnerability-stress models of depression in a self-regulatory and psychobiological context In: Handbook of depression. New York: Guilford Press (2002). 268–94.

[61] Kornadt AE Siebert JS Wahl H-W. The interplay of personality and attitudes toward own aging across two decades of later life. PLoS One. (2019) 14:e0223622. doi: 10.1371/journal.pone.0223622, PMID: 31596876 PMC6785129

[62] Melle I Friis S Hauff E Vaglum P. Social functioning of patients with schizophrenia in high-income welfare societies. Psychiatr Serv. (2000) 51:223–8. doi: 10.1176/appi.ps.51.2.223, PMID: 10655007

[63] American Psychiatric Association. Diagnostic and statistical manual of mental disorders (DSM-5®). Washington, DC: American Psychiatric Association (2013).

[64] Haslam N Reichert T Fiske AP. Aberrant social relations in the personality disorders. Psychol Psychother Theory Res Pract. (2002) 75:19–31. doi: 10.1348/147608302169526 12006197

[65] Orsmond GI Krauss MW Seltzer MM. Peer relationships and social and recreational activities among adolescents and adults with autism. J Autism Dev Disord. (2004) 34:245–56. doi: 10.1023/B:JADD.0000029547.96610.df, PMID: 15264493

[66] Ronchi L Banerjee R Lecce S. Theory of mind and peer relationships: the role of social anxiety. Soc Dev. (2020) 29:478–93. doi: 10.1111/sode.12417

[67] O’Brien EL Sharifian N. Managing expectations: how stress, social support, and aging attitudes affect awareness of age-related changes. J Soc Pers Relat. (2019) 37:986–1007. doi: 10.1177/0265407519883009, PMID: 36467591 PMC9717678

[68] Robertson DA Kenny RA. “I'm too old for that”—the association between negative perceptions of aging and disengagement in later life. Personal Individ Differ. (2016) 100:114–9. doi: 10.1016/j.paid.2016.03.096

[69] Schwartz E Ayalon L Huxhold O. Exploring the reciprocal associations of perceptions of aging and social involvement. J Gerontol Ser B. (2021) 76:563–73. doi: 10.1093/geronb/gbaa008, PMID: 31950185

[70] Montepare JM. An exploration of subjective age, actual age, age awareness, and engagement in everyday behaviors. Eur J Ageing. (2020) 17:299–307. doi: 10.1007/s10433-019-00534-w, PMID: 32904859 PMC7459006

[71] Bodner E. The importance of self-perceptions of aging in predicting late-life loneliness. Int Psychogeriatr. (2022) 34:609–12. doi: 10.1017/S1041610221002830 35000659

[72] Hu RX Li LW. Social disconnectedness and loneliness: do self-perceptions of aging play a role? J Gerontol Ser B. (2022) 77:936–45. doi: 10.1093/geronb/gbac008, PMID: 35085397 PMC9071429

[73] Lin Y Zhang B Ma Y. How do aging self-stereotypes relate to social isolation in older adults? The intervening roles of sense of coherence and cellphone use. Psychol Rep. (2022) 00332941221135483:003329412211354. doi: 10.1177/00332941221135483 36282146

[74] Moieni M Seeman TE Robles TF Lieberman MD Okimoto S Lengacher C. Generativity and social well-being in older women: expectations regarding aging matter. J Gerontol Ser B. (2021) 76:289–94. doi: 10.1093/geronb/gbaa022, PMID: 32064530 PMC7813180

[75] Sabatini S Ukoumunne OC Ballard C Collins R Kim S Corbett A. What does feeling younger or older than one’s chronological age mean to men and women? Qualitative and quantitative findings from the PROTECT study. Psychol Health. (2021) 38:324–47. doi: 10.1080/08870446.2021.1960989 34353194

[76] World Health Organization. Gender and health survey. Denmark: (2018).

