# Supplementary material for: Physical and mental health conditions account for variability in awareness of age-related changes
Source: Front Psychiatry. 2023 Jul 19;14:1152177. doi: 10.3389/fpsyt.2023.1152177 (PMC10394239; doi:10.3389/fpsyt.2023.1152177)
Supplement: Supplementary file 1 [file Table_1.docx]

**Supplementary Table 1. Descriptive statistics for demographic variables for the current study sample and participants who did not provide data**

|  | Subsample who reported AARC, and physical and mental health conditions in 2020  (n= 3787) | Subsample who did not report one or more of AARC, and physical and mental health conditions in 2020  (n= 1566) | p-value |
| --- | --- | --- | --- |
| Age; M (SD) | 67.04 (6.88) | 66.38 (6.89) | .090 |
| Women, n (%) | 2953 (78.0) | 1207 (76.9) | .374 |
| Education, n (%) |  |  |  |
| Secondary education | 512 (13.5) | 205 (13.1) | .703 |
| Post-secondary education | 438 (11.6) | 197 (12.6) |  |
| Vocational qualification | 752 (19.9) | 300 (19.1) |  |
| Undergraduate degree | 1309 (34.6) | 543 (34.6) |  |
| Post-graduate degree | 637 (16.8) | 276 (17.6) |  |
| Doctorate | 139 (3.7) | 48 (3.1) |  |
| White ethnicity, n (%) | 3557 (93.9) | 1546 (98.5) | .620 |
| Awareness of age-related gains, M (SD) | 18.31 (3.74) | 18.23 (3.67) | .500 |
| Awareness of age-related losses, M (SD) | 10.14 (3.36) | 10.27 (3.34) | .179 |

M= Mean. n = Number. AARC= Awareness of age-related changes.

**Supplementary Table 2. Number and proportion of participants having each physical and mental health condition and associations of physical and mental health conditions (predictor) with awareness of age-related gains and losses (outcomes)**

| Type of condition/predictors | n (%) of yes | AARC-gains | AARC-losses |
| --- | --- | --- | --- |
|  |  | Standardized beta (95% CI); p-value | |
| Cardiovascular disease | 1699 (44.9) | .02 (-.01; .05); .221 | .16 (.13; .19); < .001 |
| Diabetes | 138 (3.7) | .02 (-.01; .05); .341 | .05 (.02; .08); .003 |
| Mild cognitive impairment | 18 (0.5) | .02 (-.01; .05); .233 | .07 (.03; .10); <.001 |
| Parkinson’s disease | 16 (0.4) | .02 (-.02; .05); .352 | .05 (.02; .08); .003 |
| Hypothyroidism | 289 (7.8) | .01 (-.02; .04); .488 | .06 (.02; .09); .001 |
| Hyperthyroidism | 76 (2.1) | .02 (-.01; .06); .177 | .04 (.004; .07); .026 |
| Arthritic condition | 874 (23.7) | .01 (-.02; .05); .408 | .15 (.12; .18); <.001 |
| Current cancer | 67 (1.8) | .04 (.002; .07); .036 | -.01 (-.04; .02); .488 |
| Cancer in full remission – no evidence of disease | 387 (10.5) | -.0001 (-.03; .03); .998 | .05 (.02; .08); .002 |
| Osteoporosis | 246 (6.7) | -.03 (-.07; .002); .062 | .07 (.04; .10); <.001 |
| Asthma | 389 (10.5) | .01 (-.02; .05); .399 | .04 (.01; .08); .006 |
| Epilepsy | 22 (0.6) | -.01 (-.04; .02); .480 | -.01 (-.04; .02); .649 |
| Multiple sclerosis | 21 (0.6) | .01 (-.02; .04); .468 | .02 (-.02; .05); .315 |
| Motor neurone disease* | 2 (0.1) |  |  |
| Paget’s disease* | 2 (0.1) |  |  |
| Human Immunodeficiency Virus* | 2 (0.1) |  |  |
| Acquired Immunodeficiency Disease Syndrome* | 0 (0) |  |  |
| Hepatitis C virus* | 3 (0.1) |  |  |
| Huntington’ s disease* | 0 (0) |  |  |
| Depression | 747 | .01 (-.03; .04); .712 | .19 (.15; .22); <.001 |
| Mania | 18 | .01 (-.03; .04); .589 | .03 (-.002; .07); .065 |
| Anxiety disorders | 479 (14.5) | .02 (-.02; .05); .314 | .13 (.09; .16); <.001 |
| Obsessive compulsive disorder* | 9 |  |  |
| Eating disorder | 46 (1.4) | -.02 (-.05; .02); .284 | .01 (-.02; .05); .404 |
| Psychotic disorders (including schizophrenia) | 10 | .02 (-.02; .05); .295 | .04 (.01; .08); .012 |
| Personality disorder | 11 | -.03 (-.07; .01); .084 | .10 (.07; .13); <.001 |

*Note:* *For those health conditions endorsed by less than 10 people we did not run regression models. Significance level after applying Bonferroni’s correction for 13 associations is .004. AARC-gains= Awareness of positive age-related changes. AARC-losses= Awareness of negative age-related changes. All models are adjusted for age, sex, education, marital status, and working status.
